# RBMX suppresses tumorigenicity and progression of bladder cancer by interacting with the hnRNP A1 protein to regulate PKM alternative splicing

**DOI:** 10.1038/s41388-021-01666-z

**Published:** 2021-02-09

**Authors:** Qiuxia Yan, Peng Zeng, Xiuqin Zhou, Xiaoying Zhao, Runqiang Chen, Jing Qiao, Ling Feng, Zhenjie Zhu, Guozhi Zhang, Cairong Chen

**Affiliations:** 1 Center for Reproductive Medicine, Qingyuan People’s Hospital, The Sixth Affiliated Hospital of Guangzhou Medical University, Qingyuan, Guangdong China; 2 Department of Urology, Qingyuan People’s Hospital, The Sixth Affiliated Hospital of Guangzhou Medical University, Qingyuan, Guangdong China

**Keywords:** Bladder cancer, Prognostic markers

## Abstract

The prognosis for patients with metastatic bladder cancer (BCa) is poor, and it is not improved by current treatments. RNA-binding motif protein X-linked (RBMX) are involved in the regulation of the malignant progression of various tumors. However, the role of RBMX in BCa tumorigenicity and progression remains unclear. In this study, we found that RBMX was significantly downregulated in BCa tissues, especially in muscle-invasive BCa tissues. RBMX expression was negatively correlated with tumor stage, histological grade and poor patient prognosis. Functional assays demonstrated that RBMX inhibited BCa cell proliferation, colony formation, migration, and invasion in vitro and suppressed tumor growth and metastasis in vivo. Mechanistic investigations revealed that hnRNP A1 was an RBMX-binding protein. RBMX competitively inhibited the combination of the RGG motif in hnRNP A1 and the sequences flanking PKM exon 9, leading to the formation of lower PKM2 and higher PKM1 levels, which attenuated the tumorigenicity and progression of BCa. Moreover, RBMX inhibited aerobic glycolysis through hnRNP A1-dependent PKM alternative splicing and counteracted the PKM2 overexpression-induced aggressive phenotype of the BCa cells. In conclusion, our findings indicate that RBMX suppresses BCa tumorigenicity and progression via an hnRNP A1-mediated PKM alternative splicing mechanism. RBMX may serve as a novel prognostic biomarker for clinical intervention in BCa.

## Introduction

Bladder cancer (BCa) is one of the most common malignant tumors of the urinary system and has high incidence and lethality rates. In 2018, BCa was ranked 10th among the worldwide diagnoses of malignancies, with an estimated 549,000 newly diagnosed cases and 200,000 deaths annually [1]. According to the guidelines of the European Association of Urology issued in 2020 [2], BCa is a clinically heterogeneous malignancy classified into two subtypes: non-muscle-invasive bladder cancer (NMIBC) and muscle-invasive bladder cancer (MIBC). NMIBC is limited to the mucosal layer (Ta or Tis) or the submucosal layer (T1) and accounts for ~75% of newly diagnosed BCa cases; the tumor cells of MIBC invade the bladder muscle layer (T2–T4), which accounts for 25–30% of all BCa cases. NMIBC usually recurs with a 5 years, with a recurrence proportion as high as 42%, but it rarely progresses [3], while ~50% of the MIBC cases progress, and the 5-year survival rate is <10% upon distant tumor metastasis, which is the main cause of mortality for BCa patients [4–6]. However, the molecular mechanisms responsible for BCa tumorigenesis and metastasis remain largely unknown. Therefore, it is essential to explore new targets and solutions for BCa diagnosis and therapy.

The survival rate of MIBC patients remains low despite multimodal therapy. To maintain tumorigenesis and aggressiveness, cancer cells have a unique metabolic preference for converting glucose into lactate even under oxygen-rich conditions; this phenomenon is termed the Warburg effect, or aerobic glycolysis [7, 8]. The Warburg effect has received substantial attention as a novel therapeutic target in cancers, including lung cancer [9], leukemia [10], pancreatic cancer [11], and BCa [12]. Pyruvate kinase muscle isozyme (PKM) is one of the key regulators of the Warburg effect as it converts phosphoenolpyruvate (PEP) to pyruvate and generates ATP [13]. PKM1 and PKM2, produced by alternative splicing of the pre-RNA transcript of the PKM gene, play important roles in the Warburg effect. PKM1 is mainly expressed in terminally differentiated tissues to promote oxidative phosphorylation, whereas PKM2 is highly expressed in embryonic and cancer cells. An increased PKM2/PKM1 ratio has been reported in multiple cancers and has been closely associated with shorter overall survival (OS) in cancer patients [14, 15]. Understanding the regulation of PKM pre-mRNA alternative splicing is of great importance for developing cancer therapy. The splicing factor hnRNP A1, a member of the heterogeneous nuclear ribonucleoprotein (hnRNP) family that contains two RNA recognition motifs (RRM1 and RRM2), an RNA-binding RGG box (RGG), and a nuclear targeting sequence (M9) is a critical regulator of posttranscriptional gene expression that regulates mRNA splicing, metabolism, stability, localization, and translation [16, 17]. hnRNP A1 is elevated in various tumor types, including hepatocellular carcinoma [18, 19], prostate cancer [20], and multiple myeloma [21], and is involved in cancer progression by facilitating the alternative splicing of numerous gene variants [17]. For example, hnRNP A1 mediates c-Myc enhanced PKM2/PKM1 and drive alternative splicing of PKM pre-mRNA by selectively including exon 10 and excluding exon 9 [22–24]. However, whether hnRNP A1/PKM2 signaling pathway regulates the progression of BCa and the possible mechanism remains largely unknown.

RNA-binding motif protein X-linked (RBMX) is a ubiquitously expressed nuclear RNA-binding protein that is located at Xq26.3. It is composed of 391 amino acids and has a molecular weight of 43 kDa [25, 26]. Several lines of evidence have indicated that low levels of RBMX expression are associated with poor survival in people with one of various cancers. Genome sequencing has been used to identify truncation mutations of the RBMX gene in lung cancer patients, suggesting RBMX as a potential tumor suppressor [27]. Tobacco-induced mutations in RBMX may predispose smokers to developing lung cancer [28]. Most recently, RBMX was shown to maintain genomic stability and prevent cell canceration [29]. Our early experiments found that RBMX binds to hnRNP A1 proteins. Therefore, we hypothesize that RBMX regulates the alternative splicing of PKM through its interaction with the hnRNP A1 protein, resulting in suppressing BCa tumorigenicity and progression.

In this study, we aimed to identify RBMX as a tumor suppressor. We discovered that RBMX inhibited the proliferation, clone formation, migration, and invasion of BCa cells both in vitro and in vivo. Further mechanistic investigations revealed that RBMX suppresses BCa tumorigenicity and progression via hnRNP A1-mediated PKM alternative splicing. RBMX could serve as a new therapeutic target.

## Results

### Low levels of RBMX expression were associated with poor survival prognoses for BCa patients

**Fig. 1:**
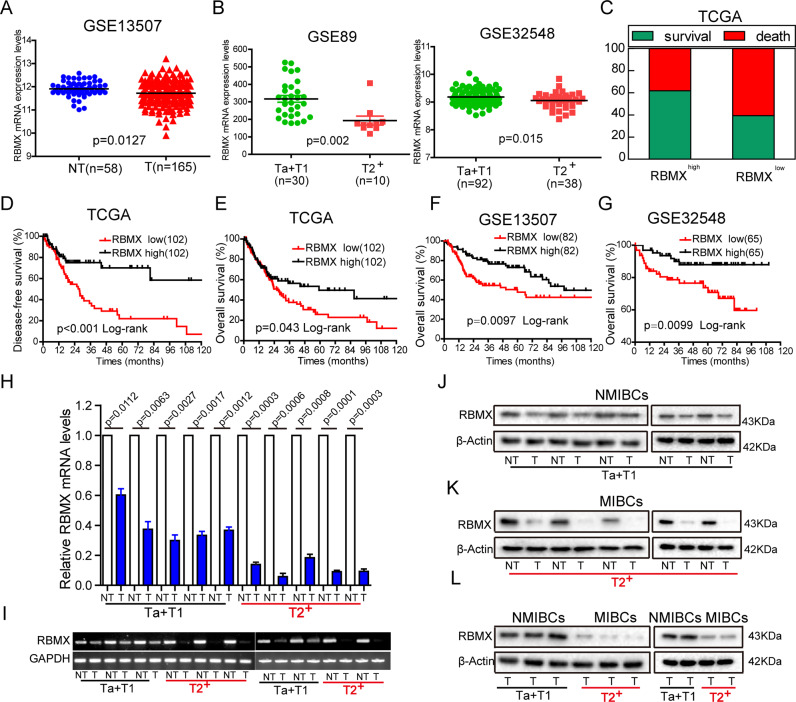
Low levels of RBMX expression were associated with poor survival prognoses for Bca patients. **A** RBMX mRNA expression levels were decreased in BCa tissues compared to those in the matched adjacent NT tissues from the GSE13507 data set. **B** Compared to that in the NMIBC tissues (Ta or T1), RBMX mRNA expression levels were decreased in the MIBC tissues (≥T2) in the GSE89 and GSE32548 data sets. **C**–**E** Compared to the rates of the patient cohort with low RBMX expression (the lower 25%), the TCGA urothelial cancer patient cohort with high RBMX mRNA expression (the upper 25%) had lower death rates and higher DFS and OS. **F**, **G** The patients with highly expressed RBMX identified in the GSE13507 and GSE32548 data sets had longer OS. **H**, **I** RBMX RNA levels in the NMIBC tissues (*n* = 5), MIBC tissues (*n* = 5), and matched adjacent NT tissues (NT) (*n* = 10) were detected by qRT-PCR and RT-PCR. **J**–**L** RBMX protein levels in the NMIBC tissues (*n* = 5), MIBC tissues (*n* = 5), and matched adjacent NT tissues (*n* = 10) were detected by western blot. Data are presented as the means ± SD.

To examine the role of RBMX in BCa carcinogenesis, we analyzed multiple data sets from multiple databases. We found that the RBMX mRNA expression levels in BCa tissues were lower than those in normal bladder tissues in the GSE13507 data set, and the difference between them was statistically significant (*P* = 0.0127) (Fig. 1A). To determine the RBMX mRNA expression difference between NMIBC tissues (Ta or T1) and MIBC tissues (≥T2), the analysis results based on the GSE89 and GSE32548 data sets showed that RBMX mRNA expression levels were decreased in the MIBC tissues, and were significant for both data sets (*P* = 0.002 and *P* = 0.015, respectively) (Fig. 1B). Similar, situations were also observed of the analysis results from the Blaveri, Sanchez-Carbayo, and Stransky bladder data sets based on the Oncomine database (Supplementary Fig. S1). Kaplan–Meier survival analyses revealed that patients with high RBMX mRNA expression (the upper 25%) had significantly lower death rates and longer disease-free survival for the TCGA urothelial cancer cohorts (Fig. 1C, D). Furthermore, the correlation of RBMX high expression with longer OS was confirmed by the analysis of the TCGA cohort (Fig. 1E), GSE13507 data set (Fig. 1F), and GSE32548 data set (Fig. 1G). A correlation analysis of clinicopathological features revealed that a low RBMX level was positively related to lymph node metastasis status (pN, *P* = 0.029) and high-grade clinical stage (*P* = 0.009) (Table 1). Additionally, RBMX mRNA expression was downregulated in the NMIBC and MIBC tissues compared to that in the matched adjacent NT tissues (Fig. 1H, I). RBMX protein expression level was consistent with the RNA expression data (Fig. 1J, K). Intriguingly, RBMX protein expression was downregulated in the MIBC tissues compared with that of the NMIBC tissues (Fig. 1L). Collectively, RBMX may serve as a marker of the progression of BCa and a prognostic marker for BCa patients.

**Table 1:** Comparison of clinical features between BUC patients with low and high RBMX levels in TCGA database.

Clinical character	Clinical groups	RBMX		*x*^2^, df	*p* value
		High (*n* = 102) (%)	Low (*n* = 102) (%)		
Age (years)	≤60	24 (23.5)	29 (28.4)	0.596, 1	0.440
	>60	78 (76.5)	73 (71.6)		
Gender	Male	70 (68.6)	75 (73.5)	0.637, 1	0.425
	Female	32 (31.4)	27 (26.5)		
Histological subtype	Papillary	37 (36.3)	27 (26.5)	2.15, 1	0.143
	Non papillary	64 (62.7)	73 (71.6)		
pT status	T2	32 (31.4)	23 (22.5)	3.47, 1	0.063
	T3–T4	58 (56.9)	76 (74.5)		
pN status	N0	65 (63.7)	51 (50.0)	4.75, 1	0.029*
	N1–N2	25 (24.5)	39 (38.2)		
Recurred/progressed	No	48 (47.1)	32 (31.4)	1.72, 1	0.190
	Yes	34 (33.3)	35 (34.3)		
Clinical stage^a^	Stage I–II	38 (37.3)	21 (20.6)	6.89, 1	0.009*
	Stage III–IV	64 (62.7)	81 (79.4)		

*BUC* urothelial carcinoma of the bladder.

^*^P < 0.05.

^a^According to the American Joint Committee on Cancer classification (Version 7) (AJCC).

### Stable overexpression of RBMX suppressed the malignant phenotypes of the BCa cells in vitro and in vivo

**Fig. 2:**
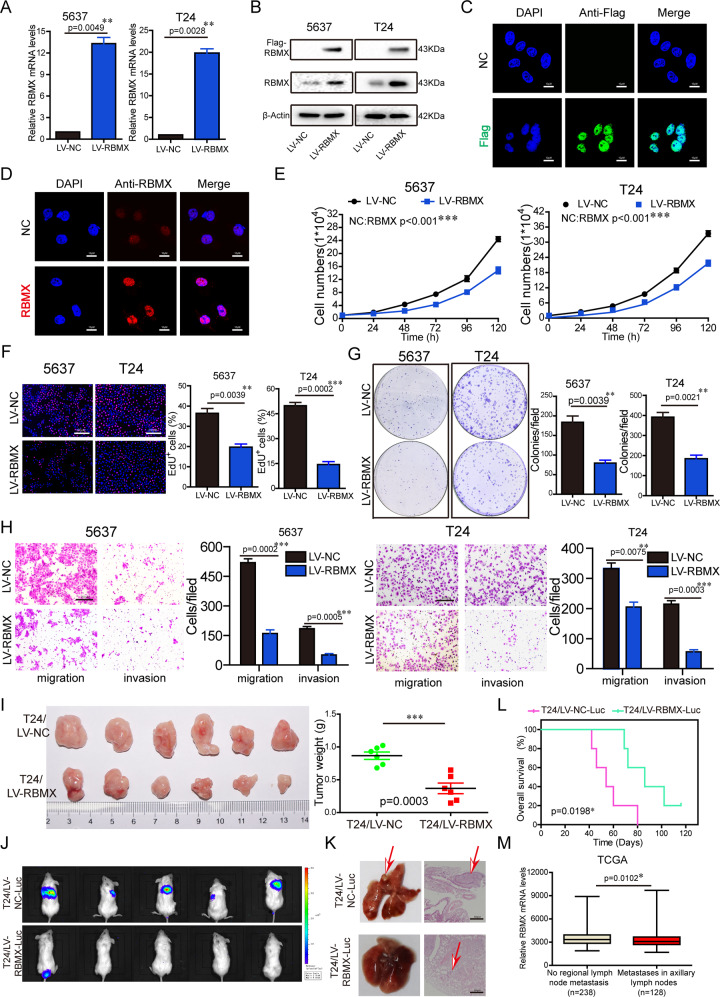
Stable overexpression of RBMX suppressed the malignant phenotypes of the BCa cells in vitro and in vivo. **A**, **B** Stable overexpression of RBMX was measured in 5637 and T24 cells by qRT-PCR and western blot. **C**, **D** The Flag-RBMX-pcDNA3.1 plasmid was stably expressed in T24 cells. Flag and RBMX were immunostained using anti-Flag and anti-RBMX antibodies, respectively. **E–H** The effects of the stable overexpression of RBMX on 5637 and T24 cell growth (**E**), proliferation (**F**), colony formation (**G**), migration, and invasion (**H**) were determined. **I** Tumor volume and weight at the end points of subcutaneous xenograft tumors formed by the T24 cells stably transfected with LV-NC or LV-RBMX into nude mice (*n* = 6 per group). **J** NOD-SCID mice were treated with via tail vein injection of T24 cells (2 × 10^6^ cells/mouse) stably transfected with LV-NC-Luc or LV-RBMX-Luc. Pulmonary metastasis was detected by an in vivo imaging system (IVIS) (*n* = 5 per group). **K** Representative images of the extent of metastasis based on lung observations and H&E staining. **L** Kaplan–Meier curves of NOD-SCID mice are shown. Data are presented as the means ± SD. **M** In TCGA urothelial cancer patient cohort, the expression of RBMX was higher in the tissues of no regional lymph node metastasis than metastases in axillary lymph nodes (*P* = 0.0102).

To investigate the role of RBMX in BCa malignant phenotypes, 5637 and T24 cells were established by lentivirus to stably overexpress RBMX or were transfected with small interfering RNAs (siRNAs) to silence RBMX. The efficiency of RBMX overexpression and knockdown was confirmed by qRT-PCR and western blot analysis (Fig. 2A, B, Supplementary Fig. 2A–C). Immunofluorescence assays showed that the RBMX protein was mainly located in the nucleus (Fig. 2C, D). Stable overexpression of RBMX significantly inhibited 5637 and T24 cell growth, cell proliferation, colony formation, migration, and invasion (Fig. 2E, H), whereas silenced of RBMX promoted 5637 and T24 cell growth, cell proliferation, colony formation, migration, and invasion (Supplementary Fig. 2D–G). These results demonstrate that RBMX is a negative regulator of BCa malignant phenotypes.

We next examined the effect of RBMX on the tumourigenicity of BCa in vivo by using xenograft model. As shown in Fig. 2I, the volume of tumors formed by the T24/LV-RBMX cells was smaller than that of the tumors derived from the T24/LV-NC cells. The weight of tumors was consistent with the volume of tumors. We next evaluated the effect of RBMX on the metastasis of T24 cells. The results showed that the metastatic nodules developed in mouse lungs were suppressed after tail vein injection with T24/LV-RBMX-Luc compared with those that developed after injection with T24/LV-NC-Luc (Fig. 2J). H&E staining was used to confirm the lung metastatic nodules (Fig. 2K). Furthermore, a Kaplan–Meier analysis showed that the mice injected with T24/LV-RBMX-Luc had longer survival times (Fig. 2L). Finally, we confirmed this by the analysis of the TCGA cohort, which the expression of RBMX was higher in the tissues of no regional lymph node metastasis than metastases in axillary lymph nodes (*P* = 0.0102) (Fig. 2M). Collectively, these data suggested that stable overexpression of RBMX suppressed the malignant phenotypes of the BCa cells in vitro and in vivo.

### RBMX interacted with the RGG box in the hnRNP A1 protein

**Fig. 3:**
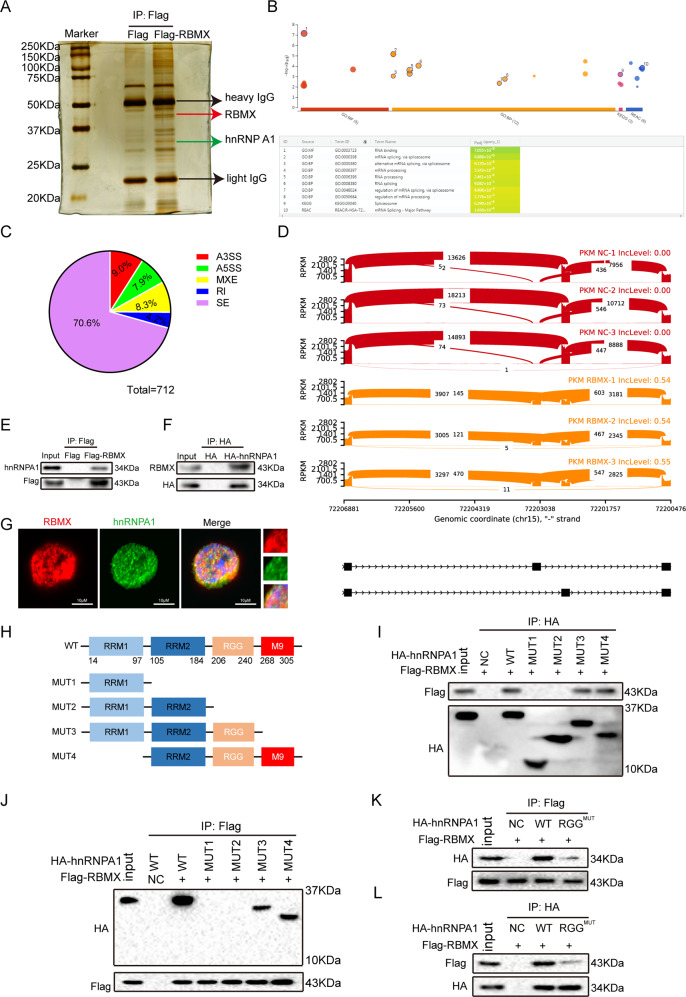
RBMX interacted with the RGG box in hnRNP A1. **A** Proteins that interacted with RBMX were identified by silver staining and mass spectrometry. **B** The RBMX-binding protein was analyzed by g: Profiler (https://biit.cs.ut.ee/gprofiler/). **C** RBMX-regulated alternative splicing events (A3SS/A5SS, alternative 3′/5′ splice sites; MXE mutually exclusive exons, RI retained introns, SE skipped exons) were detected by RNA-seq of the T24 cells. **D** PKM pre-mRNA splicing was regulated by RBMX overexpression. **E**, **F** The Flag-RBMX or HA-hnRNP A1 plasmids was transfected into HEK293T cells, Flag-RBMX complexes were coimmunoprecipitated by anti-Flag antibody, and then hnRNP A1 was detected by anti-hnRNP A1 antibody (**E**); HA-hnRNP A1 complexes were coimmunoprecipitated by anti-HA antibody and then detected by anti-RBMX antibody (**F**). **G** RBMX (red) and hnRNP A1 (green) colocalization in the T24 cells was detected by confocal microscopy. **H** Diagram of the wild-type hnRNP A1 and mutation constructs with different domains. **I**, **J** Wild-type hnRNP A1 and different mutation constructs together with the Flag-RBMX plasmid were transfected into HEK293T cells, anti-HA antibody was used for the coimmunoprecipitation, and Flag-RBMX was detected using anti-Flag antibody (**I**); anti-Flag antibody was used to coimmunoprecipitate, and wild-type hnRNP A1 and different mutation constructs were detected using anti-HA antibody (**J**). **K**, **L** Wild-type hnRNP A1 and RGG^MUT^ plasmids together with the Flag-RBMX plasmid were co-transfected into HEK293T cells; the interactions of RBMX with hnRNP A1 RGG^MUT^ were determined as described in (**I**)(**K**) and (**J**)(**L**).

RBMX has been identified as a suppressor gene that inhibits tumorigenesis and tumor progression [30]. RBMX and its binding proteins were pulled down using Flag antibody immunoprecipitation, and the proteins that interacted with RBMX were identified (Fig. 3A). Then, a total of 67 proteins that interact with the RBMX protein were identified by MS analysis (Supplementary Table 1).

To characterize the functional roles of these RBMX-interacting proteins, their biological processes and protein–protein interaction networks were analyzed using bioinformatics analyses [31]. A protein–protein interaction assay combined with a Gene Ontology annotation assay showed that the 67 proteins were mainly enriched in the RNA splicing category (Fig. 3B, Supplementary Table 2), suggesting that the RBMX protein might regulate cellular RNA splicing. Among the 67 proteins that interact with RBMX protein, the splicing factor hnRNP A1 was of particular interest because hnRNP A1 had a higher MS score among our identified splicing regulators (Supplementary Fig. 3A, B, Supplementary Table 1) and is the key regulator of PKM pre-mRNA alternative splicing in cancer [24]. Furthermore, we performed high-throughput RNA-seq of WT and RBMX-overexpressing T24 cells, and 712 RBMX-regulated alternative splicing events were identified (Fig. 3C). Interestingly, we found that RBMX-regulated PKM pre-mRNA splicing, inhibited PKM2 isoform formation and promoted PKM1 isoform formation (Fig. 3D). On the contrary, hnRNP A1 protein regulated PKM pre-mRNA splicing promoting the formation of the PKM2 isoform [22]. Therefore, we speculate that RBMX and hnRNP A1 have antagonistic effects on the regulation of PKM pre-mRNA splicing.

To confirm the interaction between RBMX and hnRNP A1, coimmunoprecipitation (co-IP) and immunofluorescence experiments were performed. As the RBMX protein was tagged with Flag and the hnRNP A1 protein was tagged with HA, we used Flag antibody or HA antibody to immunoprecipitate RBMX or hnRNP A1 and then detected hnRNP A1 and RBMX by western blot. As shown in Fig. 3E, F, hnRNP A1 or RBMX was detected with Flag- or HA-immunoprecipitated proteins but not in input. In addition, immunofluorescence images showed that RBMX colocalized with hnRNP A1 in the nucleus (Fig. 3G). These results indicate that RBMX binds to the hnRNP A1 protein. On the basis of these data, we hypothesize that RBMX may be involved in the hnRNP A1-mediated pre-mRNA alternative splicing of the PKM gene.

hnRNP A1 consists of RRM1, RRM2, RGG, and M9 motifs. To investigate which motifs interact with RBMX protein, we generated hnRNP A1 truncated constructs with a C-terminal HA-tag and co-expressed them with RBMX-Flag in HEK293T cells (Fig. 3H). Only the constructs containing the RGG box, but not the other regions in hnRNP A1, retained the ability to interact with RBMX, indicating that the RGG box in hnRNP A1 is essential for RBMX binding (Fig. 3I, J).The RGG motif mediates not only the binding of RGG motif proteins to RNA but also the interaction of RGG motif proteins with other proteins [32]. We mutated the RGG box (Arg-Gly-Gly) in hnRNP A1 to be an AAA box (Ala-Ala-Ala) to construct the hnRNP A1 AAA mutant (RGG^MUT^). We found that the RBMX protein strongly bound to wild-type hnRNP A1 (WT) but weakly bound to RGG^MUT^ (Fig. 3K, L), indicating that the mutation of the RGG box disrupted the interaction of hnRNP A1 with the RBMX protein.

### RBMX antagonized the hnRNP A1-induced aggressive phenotype of the BCa cells

Using the Oncomine database, we found that hnRNP A1 mRNA expression was upregulated in BCa tissue compared with that in normal bladder tissue in different data sets (Supplementary Fig. 4A–D). In addition, our results showed that hnRNP A1 mRNA expression was upregulated in the NMIBC and MIBC tissues compared to that in the matched adjacent NT tissues (Supplementary Fig. 4E). hnRNP A1 protein expression level was consistent with the RNA expression data. (Supplementary Fig. 4F, G). However, hnRNP A1 protein expression was no significant difference in the MIBC tissues compared with that of the NMIBC tissues (Supplementary Fig. 4H). Furthermore, there was no correlation between the expression of RBMX and hnRNP A1 in tumor tissues of BCa patients (*R* = 0.0690, *P* = 0.4635) (Supplementary Fig. 4I). To investigate the influences of hnRNP A1 on cancer phenotypes, the expression of hnRNP A1 was silenced (Supplementary Fig. 4J). Silencing hnRNP A1 suppressed BCa cell growth, colony formation, migration, and invasion (Supplementary Fig. 4K–M).

**Fig. 4:**
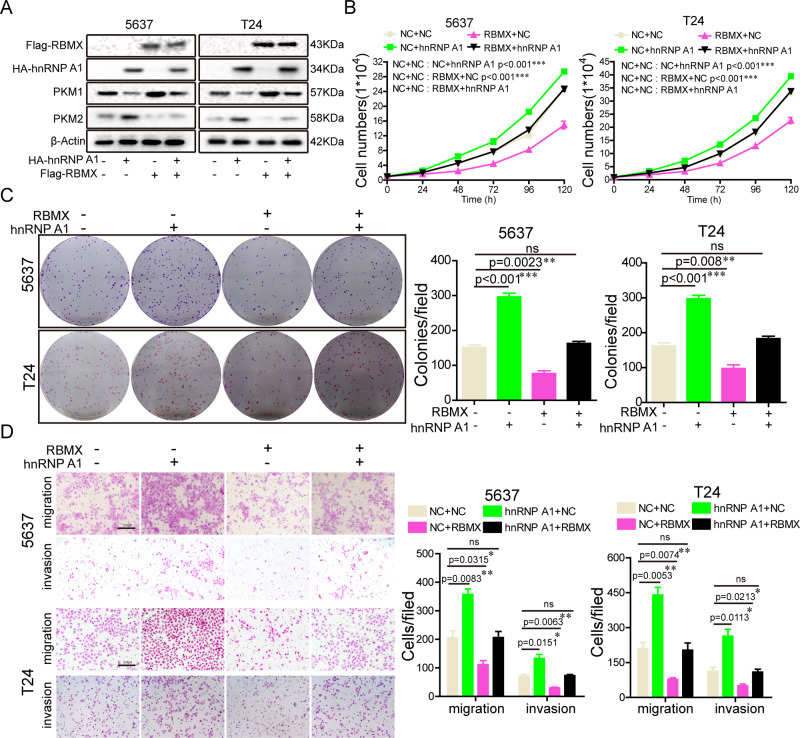
RBMX antagonized the hnRNP A1-induced aggressive phenotype of the BCa cells. Flag-RBMX, HA-hnRNP A1, or Flag-RBMX plasmid together with the HA-hnRNP A1 plasmid were transfected into 5637 and T24 cells, and the indicated protein expression (**A**), cell growth (**B**), colony formation (**C**), migration, and invasion (**D**) were determined. Data are presented as the means ± SD.

We next sought to determine whether RBMX affects hnRNP A1 functions, the Flag-RBMX or HA-hnRNP A1 plasmids were co-transfected together into 5637 and T24 cells. The western blot analysis results showed the expression of different proteins in combination experiments (Fig. 4A). We also found that RBMX antagonized the enhancement of cell growth, colony formation, migration, and invasion had been induced by hnRNP A1 overexpression (Fig. 4B–D). These pieces of evidence indicated that RBMX attenuated the oncogenic properties of hnRNP A1 by binding to it.

### RBMX hindered the binding of hnRNP A1 to the sequences flanking exon 9 of PKM by competitively binding to the RGG box in hnRNP A1

**Fig. 5:**
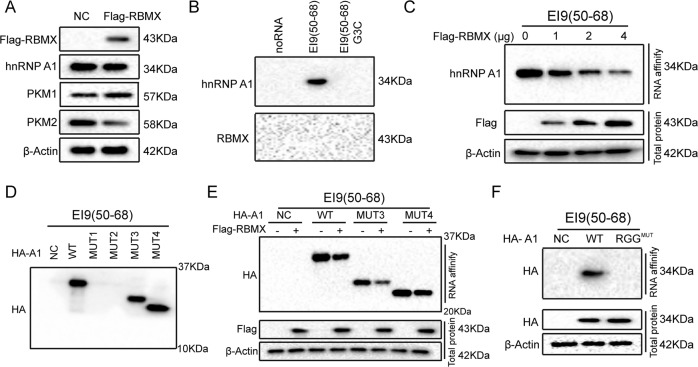
RBMX hindered the binding of the RGG box in hnRNP A1 to the sequences flanking exon 9 of PKM. **A** The Flag-RBMX plasmid was transfected into T24 cells, and the protein levels of RBMX, hnRNP A1, PKM1, and PKM2 were detected. **B** T24 cell nuclear extracts were affinity-purified using the indicated biotin-labeled RNAs, and then the eluted proteins were indicated by anti-RBMX and anti-hnRNP A1 antibodies. **C** Flag-RBMX plasmid at the specified doses was transfected into T24 cells, and the hnRNP A1 expression level was detected after affinity purification using biotin-labeled RNA EI9 (50–68). **D** Wild-type hnRNP A1 and MUT1–MUT4 were transfected into T24 cells, and the indicated protein levels were determined after affinity purification using biotin-labeled RNA EI9 (50–68). **E** Wild-type hnRNP A1 or hnRNP A1 MUT3–MUT4 plasmids together with the Flag-RBMX plasmid were transfected into T24 cells, and the indicated protein levels were measured after affinity purification using biotin-labeled RNA EI9 (50–68). **F** Wild-type hnRNP A1 or RGG^MUT^ was transfected into T24 cells, and the indicated protein levels were determined after affinity purification using biotin-labeled RNA EI9 (50–68).

hnRNP A1 is a critical determinant of the metabolic phenotype in cancer cells by promoting aerobic glycolysis [22, 24, 33]. hnRNP A1 has been proven to promote the formation of the PKM2 isoform by binding to the intronic UAGGGC sequences flanking exon 9 (EI9) [24]. We found that exogenous RBMX did not change the level of hnRNP A1 protein but upregulated PKM1 expression and downregulated PKM2 (Fig. 5A). To further investigate the role of RBMX in the ability of hnRNP A1 bind to PKM EI9, RNA affinity chromatography using 5′ biotin-labeled EI9 (50–68) containing a UAGGGC sequence and EI9 (50–68) G3C (mutation of the G3 nucleotide to C in UAGGGC), which binds to hnRNP A1, was performed as previously described [34]. Strong binding of hnRNP A1 to the EI9 (50–68) sequence of PKM was observed, whereas the mutation in the G3 nucleotide to C in EI9 (50–68) resulted in failure binding to hnRNP A1 (Fig. 5B, upper panel). We found that the RBMX did not directly bind to the mRNA sequences of PKM (Fig. 5B, lower panel). Furthermore, RBMX blocked the binding of hnRNP A1 to EI9 (50–68) of PKM in a dose-dependent manner (Fig. 5C).

To investigate which domain in hnRNP A1 can bind to EI9 (50–68) of PKM, truncated constructs of hnRNP A1 (MUT1–MUT4) with an HA-tag were transfected into T24 cells. We found that the truncated constructs of hnRNP A1 can bind to EI9 (50–68) only when it contains the RGG box, indicating that the RGG box in hnRNP A1 is essential for binding to the EI9 (50–68) sequences of PKM (Fig. 5D). Moreover, RBMX blocked the binding of the RGG box in hnRNP A1 to the EI9 (50–68) sequences of PKM (Fig. 5E). We also found that hnRNP A1 lost the ability to bind to the EI9 (50–68) sequences of PKM when the RGG box was mutated (Fig. 5F). Collectively, our results demonstrate that RBMX blocked the binding of hnRNP A1 to the mRNA sequences of PKM by competitively binding to the RGG box of hnRNP A1.

We next to determine whether RGG^MUT^-hnRNP A1 affects PKM alternative splicing, cancer phenotypes, and cellular glycolysis. After knocking down hnRNP A1, wild-type hnRNP A1 (wt-hnRNP A1), RGG^MUT^-hnRNP A1 or wt-hnRNP A1 plasmid together with the RGG^MUT^-hnRNP A1 plasmid were transfected into T24 BCa cells (Supplementary Fig. 5A). Compared with the overexpressed wt-hnRNP A1 plasmid, RGG^MUT^-hnRNP A1 could not regulate PKM alternative splicing (Supplementary Fig. 5B) and also could not promote cell proliferation (Supplementary Fig. 5C), colony formation (Supplementary Fig. 5D), migration, invasion (Supplementary Fig. 5E), glucose uptake (Supplementary Fig. 5F) and lactic acid production (Supplementary Fig. 5G). These results preliminarily indicate that RGG domain is the key domain of hnRNP A1 in cell function.

### RBMX inhibited aerobic glycolysis through hnRNP A1-dependent PKM splicing

**Fig. 6:**
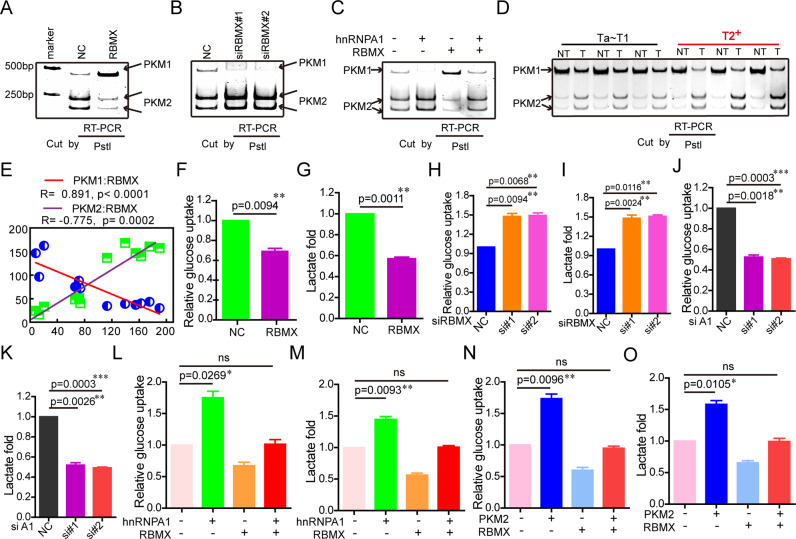
RBMX inhibited aerobic glycolysis through hnRNP A1-dependent PKM splicing in the T24 cells. **A**, **B** Flag-RBMX plasmids (**A**) or the anti-RBMX siRNAs (**B**) were transfected into T24 cells, and PKM splicing was performed using PstI. **C** Flag-RBMX, HA-hnRNP A1, or Flag-RBMX plasmid together with the HA-hnRNP A1 plasmid were transfected into T24 cells, and then, PKM splicing was induced using PstI. **D** PKM splicing was performed in the NMIBC tissues, MIBC tissues, and matched adjacent normal tissues. **E** The mRNA levels of PKM1 and PKM2 were positively and negatively correlated with the RBMX mRNA levels in the NMIBC tissues (*n* = 3), MIBC tissues (*n* = 3), and matched adjacent normal tissues (*n* = 6). **F**–**K** Flag-RBMX plasmid (**F**, **G**), RBMX siRNAs (**H**, **I**), or hnRNP A1 siRNAs (**J**, **K**) were transfected into T24 cells, and then, the glucose uptake and lactate production were measured. **L**, **M** Flag-RBMX, HA-hnRNP A1, or Flag-RBMX together with the HA-hnRNP A1 plasmid were transfected into T24 cells, and then, the glucose uptake and lactate production were measured. **N**, **O** Flag-RBMX, HA-PKM2, or Flag-RBMX plasmid together with the HA-PKM2 plasmid were transfected into T24 cells, and then, the glucose uptake and lactate production were measured. Data are presented as the means ± SD.

We confirmed that RBMX blocked the binding of hnRNP A1 to EI9 of the PKM gene described above. Therefore, the effects of RBMX on PKM splicing were further studied by RT-PCR followed by restriction digestion to measure the PKM1 and PKM2 levels as previously described [33]. Our results showed that RBMX increased the level of PKM1 mRNA and decreased the level of PKM2 mRNA (Fig. 6A). By contrast, RBMX silencing resulted in the opposite phenomenon (Fig. 6B). RBMX attenuated the increase in PKM2 mRNA and the decrease in PKM1 mRNA that had been induced by overexpression of hnRNP A1 (Fig. 6C). Furthermore, PKM2 mRNA levels increased and PKM1 mRNA levels decreased in the BCa tissues compared to those in the corresponding bladder NT tissues (Fig. 6D). The mRNA levels of PKM1 and PKM2 were positively (*R* = 0.891, *P* < 0.0001) and negatively (*R* = −0.775, *P* = −0.0002) correlated with the RBMX mRNA levels in the sample tissues, respectively (Fig. 6E).

As a key factor in aerobic glycolysis, the M2 splice isoform of pyruvate kinase (PKM2) can increase glucose uptake and lactate production [35, 36]. Consistently, stable overexpression of RBMX decreased glucose uptake (Fig. 6F, Supplementary Fig. 6A) and lactate production (Fig. 6G, Supplementary Fig. 6B) in T24 and 5637 cells, respectively, whereas silencing of RBMX led to the opposite results (Fig. 6H, I, Supplementary Fig. 6C, D). In addition, silencing hnRNP A1 decreased glucose uptake and lactate production (Fig. 6J, K, Supplementary Fig. 6E, F). Fortunately, the expression of RBMX attenuated the enhanced glucose uptake and lactate production induced by hnRNP A1 overexpression (Fig. 6L, M, Supplementary Fig. 6G, H). As expected, overexpression of PKM2 increased glucose uptake and lactate production, and RBMX attenuated the enhanced glucose uptake and lactate production induced by PKM2 overexpression (Fig. 6N, O, Supplementary Fig. 6I, J). Taken together, our results showed that RBMX inhibited BCa cell aerobic glycolysis by suppressing hnRNP A1-mediated PKM splicing.

### RBMX counteracted the PKM2 overexpression-induced aggressive phenotype of the BCa cells

**Fig. 7:**
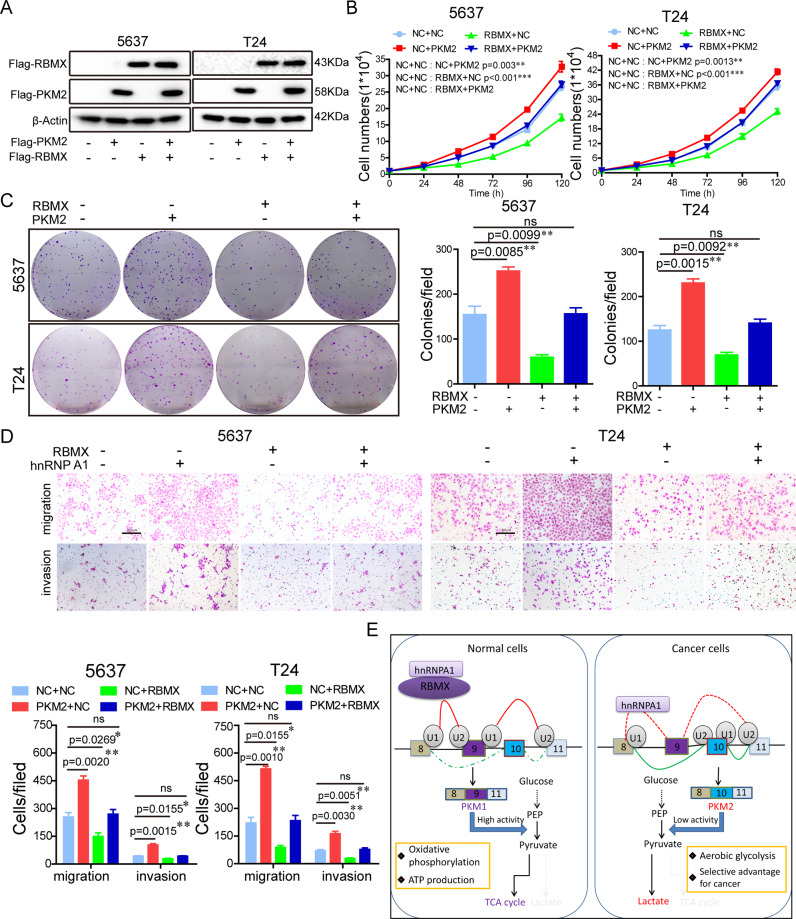
RBMX counteracted the PKM2 overexpression-induced aggressive phenotype. **A**–**D** Flag-RBMX, HA-PKM2, or Flag-RBMX plasmid together with the HA-PKM2 plasmid were transfected into 5637 and T24 cells, and the indicated protein expression (**A**), cell growth (**B**), colony formation (**C**), and migration and invasion (**D**) were detected. **E** A schematic model of the mechanism underlying the role of RBMX in BCa aerobic glycolysis through regulating PKM alternative splicing mediated by hnRNP A1.

Recent data indicated that high expression levels of PKM2 contributed to the aerobic glycolysis and promoted the proliferation of the BCa cells [37]. To determine the relationship between RBMX and PKM2 in the BCa cell aggressive phenotype, Flag-RBMX, Flag-PKM2 or Flag-RBMX plasmid together with the Flag-PKM2 plasmid were co-expressed in 5637 and T24 cells (Fig. 7A). We found that overexpression of PKM2 promoted 5637 and T24 cell growth, colony formation, migration and invasion, whereas RBMX attenuated this these effects caused by PKM2 overexpression (Fig. 7B–D). In summary, these findings indicated that RBMX suppressed the BCa cell aggressive phenotype by inhibiting PKM2 formation.

## Discussion

The malignant progression from NMIBC to MIBC is a principal cause of mortality for BCa patients, and treatments for MIBC are currently limited and unsatisfactory. Therefore, elucidation of the molecular mechanisms that drive malignant progression in this disease might lead to clinical prevention and effective therapies for malignant BCa patients. Recently, several molecules associated with the malignant progression of BCa have been studied, including PTBP1 [37], DANCR [38], and hnRNP K [39]. In this study, we reported that RBMX was markedly downregulated in the BCa specimens, especially the MIBC specimens. We found that the expression of RBMX was negatively associated with poor clinical prognosis. The overexpression of RBMX inhibited the proliferation and metastasis of BCa cells in vitro and in vivo. Mechanistically, RBMX competitively binds to the RGG box in hnRNP A1 and antagonizes hnRNP A1-mediated regulation of PKM splicing by blocking the binding of the RGG motif in hnRNP A1 to the sequences flanking the PKM exon 9, resulting in the downregulation of PKM2 and the upregulation of PKM1.

RBMX is a ubiquitously expressed nuclear RNA-binding protein located on the X chromosome. As a splicing factor, RBMX regulates RNA splicing and transcription [40, 41], and plays an important role in DNA damage repair [42] and genome integrity maintenance [29]. A network analysis of gene expression data taken from thousands of tumors indicated that RBMX is a key switch that is closely linked to important cancer drivers [43]. Increased levels of RBMX expression are associated with a favorable outcome for patients with endometrial cancer [44]. Consistent with these findings, our bioinformatics analysis results which based on several publicly available expression profiling data sets (Oncomine, GEO, and TCGA) demonstrated that RBMX expression level was negatively correlated with lymph node metastasis status and clinical stage. Additionally, overexpression of RBMX suppressed BCa cell growth, colony formation, migration, and invasion in vitro and in vivo. Nevertheless, the molecular mechanisms underlying RBMX-suppressed tumorigenicity and progression of BCa are still unknown.

Surprisingly and intriguingly, we discovered that RBMX protein mainly interacts with RNA-binding proteins, indicating that it may play an important role in cancer progression by regulating RNA splicing and processing [45–47]. For instance, hnRNP family proteins (hnRNP A1, I, K, and U) regulate tumor development and progression by mediating RNA splicing and processing [19, 48–51]. In our study, 6 hnRNP proteins were found to interact with RBMX protein, suggesting that RBMX inhibited cancer progression by participating in the regulation of RNA splicing and processing. Particularly, we discovered that RBMX binds to the RGG box in hnRNP A1, thus blocking the binding of the RGG box in hnRNP A1 to the EI9 sequences of the PKM gene, as verified by hnRNP A1 mutation assays and RNA pull-down assays, and subsequently inhibiting the formation of the PKM2 isoform.

It is well known that cancer cells, in contrast to normal cells, rely on aerobic glycolysis to generate the energy needed for cellular processes rather than mitochondrial respiration regardless of oxygen available [52]. The inhibition of the glycolytic pathway is considered to be a novel strategy for cancer therapy [53]. PKM is the rate-limiting enzyme for the last step of glycolysis. The PKM gene consists of 12 exons, of which exons 9 and 10 are alternatively spliced to give rise to the PKM1 and PKM2 isoforms [34]. PKM2 upregulation and PKM1 downregulation play important roles in metabolism and growth by enhancing the Warburg effect in cancer [54]. The critical switch of the PKM1 to the PKM2 isoform is regulated by hnRNP A1 and promotes cancer progression in multiple malignancies [23, 24]. This was confirmed in our study to occur in BCa. Furthermore, RBMX blocked hnRNP A1-dependent PKM2 isoform formation, resulting in the formation of the PKM1 isoform, which reduced glucose uptake and lactate production, thereby inhibiting the proliferation of BCa cells. In general, our study suggest a novel mechanism by which RBMX inhibits BCa cell proliferation and migration through hnRNP A1-mediated PKM alternative splicing and inhibits PKM2 formation driven by RBMX combined with the RGG box in hnRNP A1.

Interestingly, besides its well established role in aerobic glycolysis, PKM2 directly regulates gene transcription. Yang et al. [55] found that PKM2 can regulate the expression of MYC and CCND1 as a transcription factor and played its non-metabolic transcriptional functions. This was confirmed in our study. Overexpression of PKM2 could indeed up-regulate the expression of MYC and CCND1. However, the transcriptional regulatory function of PKM2 was inhibited while both PKM2 and RBMX were co-overexpressed (the data unpublished). Thus, our results preliminarily indicate that PKM2 enzyme activity and transcriptional activity were inhibited under the action of RBMX. Further investigation of the detailed molecular mechanisms are currently under way.

Recently, there is a growing demand to develop therapeutic approaches which target aberrant RNA splicing events [56]. Small molecules, such as E7107, have been designed to target the RNA core spliceosome components, and impair cancer-related pre-mRNA splicing in a dose- and time-dependent manner [57]. In addition, oligonucleotide-based therapies which target specific pathologic splicing events show promising. In fact, clinical trial for patients with Duchenne muscular dystrophy which investigate the modulation of RNA splicing events as a potential disease therapy have been established (Number NCT: NCT00844597) [58]. In the future, inhibition of cancer proliferation and metastasis via small molecules that specifically target hnRNP A1 and specific oncogenic RNA splicing events might be a potential therapeutic methods for BCa.

In summary, the present study identifies a newly negative regulator of aerobic glycolysis, RBMX, which is significantly downregulated in BCa tissues, and was negatively correlated with tumor stage, histological grade and patient prognosis. RBMX suppresses BCa tumorigenicity and progression via an hnRNP A1-mediated PKM alternative splicing mechanism (Fig. 7E). These observations advance our understanding of RBMX/hnRNP A1/PKM2 axis in BCa and suggest that RBMX may have utility as a novel prognostic biomarker and or/therapeutic target for BCa.

## Materials and methods

### Tissue samples, experimental animal models, and cell culture

BCa tissues and matched normal bladder tissues were collected from patients treated at Qingyuan People’s Hospital. These patients were selected on the basis of clear pathological diagnosis and no preoperative anticancer treatment. Experimental animal models and cell culture details are provided in the Supplementary Materials.

### Quantitative reverse-transcription PCR (qRT-PCR)

Total RNA was extracted from cell lines using TRIzol reagent (Invitrogen, USA) according to the manufacturer’s instructions. Detailed information is provided in the Supplementary Materials. The primer sequences used in this study are listed in Supplementary Table 3.

### Western blot analysis

Western blot were performed according to the manufacturer’s instructions. Detailed information is provided in the Supplementary Materials.

### Immunofluorescence

Immunofluorescence were performed according to the manufacturer’s instructions. Detailed information is available in the Supplementary Materials.

### Plasmid constructs

Full-length RBMX, hnRNP A1, and PKM2 were amplified using PCR and cloned into a pcDNA3.1 (+) vector (Invitrogen, USA) using TaKaRa Primer STAR Max DNA polymerase. Mutants of hnRNP A1 (MUT1–MUT 4) and mutant hnRNP A1 RGG box (hnRNP A1 AAA box) were produced by PCR using a Mut Express II Fast Mutagenesis Kit V2 (Vazyme, C214). The primers used for the plasmid constructs are listed in Supplementary Table 3.

### Lentivirus production and the generation of stable cell lines

Lentivirus production and the generation of stable cell lines were performed according to the manufacturer’s instructions. Detailed methods are provided in the Supplementary Materials.

### RNA interference

All the specific siRNAs and negative control siRNA were synthesized by GenePharma (Suzhou, China) based on the following sequences: RBMX siRNA#1, 5′-CGGAUAUGGUGGAAGUCGATT-3′; RBMX siRNA#2, 5′- UCAAGAGGAUAUAGCGAUATT-3′; hnRNP A1 siRNA#1, 5′-CAGCUGAGGAAGCUCUUCATT-3′; hnRNP A1 siRNA#2, 5′-GCUGUGUAAAGUUAGUCUATT-3′; and NC siRNA, 5′-GUACCGCACGUCAUUCGUAUC-3′. T24 cell transfection was performed in 6-well plates using RNAi MAX (Invitrogen, USA). Forty-eight hours after transfection, the cells were harvested to perform the analyses.

### Cell growth and proliferation assay

Detailed methods are provided in the Supplementary Materials.

### Cell colony formation assay

Detailed methods are provided in the Supplementary Materials.

### Cell migration and invasion assays

Detailed methods are provided in the Supplementary Materials.

### In vivo xenograft tumor model

For animal studies, blinding was not used. The T24 cells overexpressing RBMX (T24/LV-RBMX, T24/LV-RBMX-Luc) and the corresponding negative control (T24/LV-NC, T24/LV-NC-Luc) were used in these assays. Both of subcutaneous tumor model and spontaneous lung metastasis model were established. Detailed methods are provided in the Supplementary Materials.

### Coimmunoprecipitation and mass spectrometry

co-IP was performed as previously described [59] with some modifications. Detailed procedure is provided in the Supplementary Materials.

### RNA-seq assays and data analysis

Total RNA of T24 cells (stably transfected with RBMX or empty vector) was extracted by TRIzol reagent. RNA-seq assays was performed by Novelbio Company (Shanghai, China). All samples were sequenced by an Illumina HiSeqX sequencing system with a paired-end 150 bp read length. RNA-seq reads were mapped to the human genome (GRCh37, primary assembly). Differential alternative splicing was quantified using the rMATS program in a HISAT2 output bam file [60]. To find distinct differential splicing events, the filter condition (FDR < 0.05, and ΔPSI ≥ 0.2) was executed. The RNA-seq data for this study have been deposited in the Gene Expression Omnibus (accession code GSE150548).

### RNA affinity purification

Based on previous study [34], the 5′-biotin-labeled RNAs of PKM exon 9 were synthesized: EI9 (50–68), biotin-AGGUAGGGCCCUAAGGGCA, and EI9 (50–68, G3C), biotin-AGGUACGGCCCUAAGGGCA. The RNA affinity purification experiment was performed as previously described [24, 61] with some modifications. Detailed procedure is provided in the Supplementary Materials.

### RT-PCR and PKM splicing assays

According to previous study [24], PKM RT-PCR and PKM splicing assays were carried out. Detailed procedure is provided in the Supplementary Materials. The primers used for PKM RT-PCR are listed in Supplementary Table 3.

### Measurement of glucose uptake and lactate production

Detailed methods are provided in the Supplementary Materials.

### Statistical analysis

All sample sizes were equal to or greater than the general sample size recommended in the previous report. GraphPad Prism 5.0 (GraphPad Software, USA) was mainly used for the data analysis. The Chi-squared test was used to analyze the differences in clinical characteristics. Survival analysis was determined using Kaplan–Meier estimation and log-rank tests. Two-tailed Student’s *t* test (unpaired or paired) was applied to compare two groups and identify significant differences. The data are presented as the means ± standard deviation of at least three independent experiments. *P* < 0.05 was considered to be significant (*P < 0.05; **P < 0.01; ***P < 0.001).

## Supplementary information

Supplementary Figure 1

Supplementary Figure 2

Supplementary Figure 3

Supplementary Figure 4

Supplementary Figure 5

Supplementary Figure 6

Supplementary Figures Legends

Supplementary Table 1

Supplementary Table 2

Supplementary Table 3

Supplementary Materials

## Data Availability

RNA-seq data supporting the results of this study have been deposited in the NCBI GEO database (https://www.ncbi.nlm.nih.gov/geo/query/acc.cgi?acc=GSE150548), under accession number GSE150548.
