# Supplementary figures and images for: RBMX suppresses tumorigenicity and progression of bladder cancer by interacting with the hnRNP A1 protein to regulate PKM alternative splicing
Source: Oncogene. 2021 Feb 9;40(15):2635–50. doi: 10.1038/s41388-021-01666-z (PMC8049873; doi:10.1038/s41388-021-01666-z)

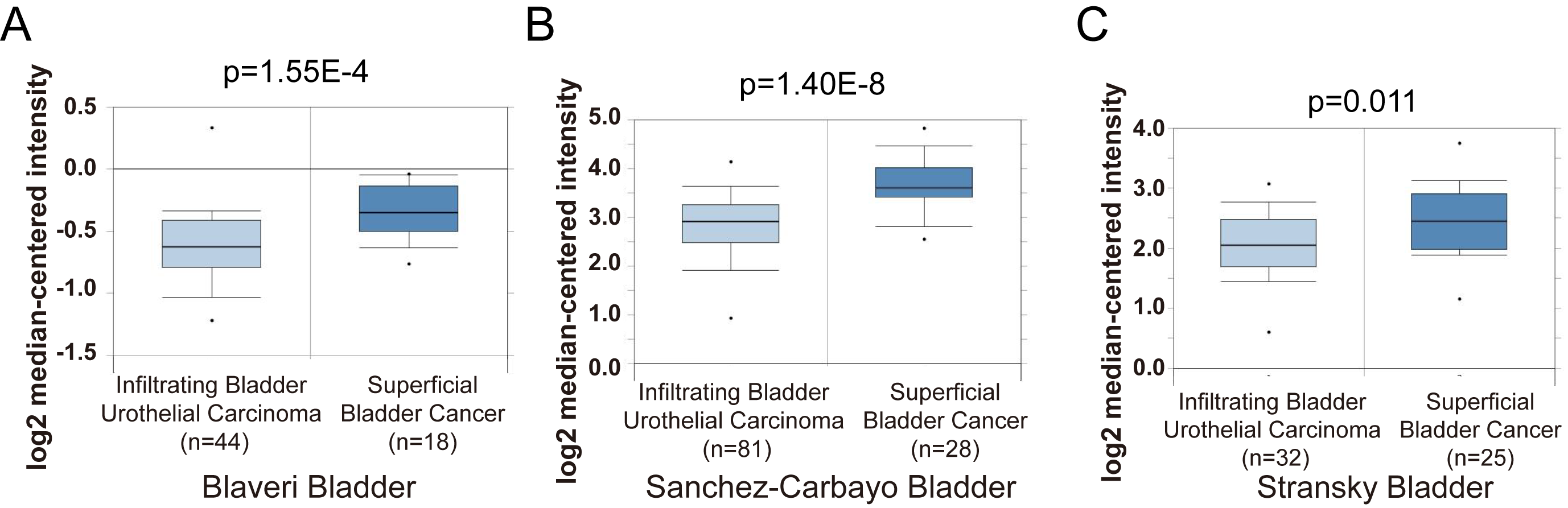

Supplement: Supplementary file 1 — Supplementary Figure 1 [file 41388_2021_1666_MOESM1_ESM.tif]

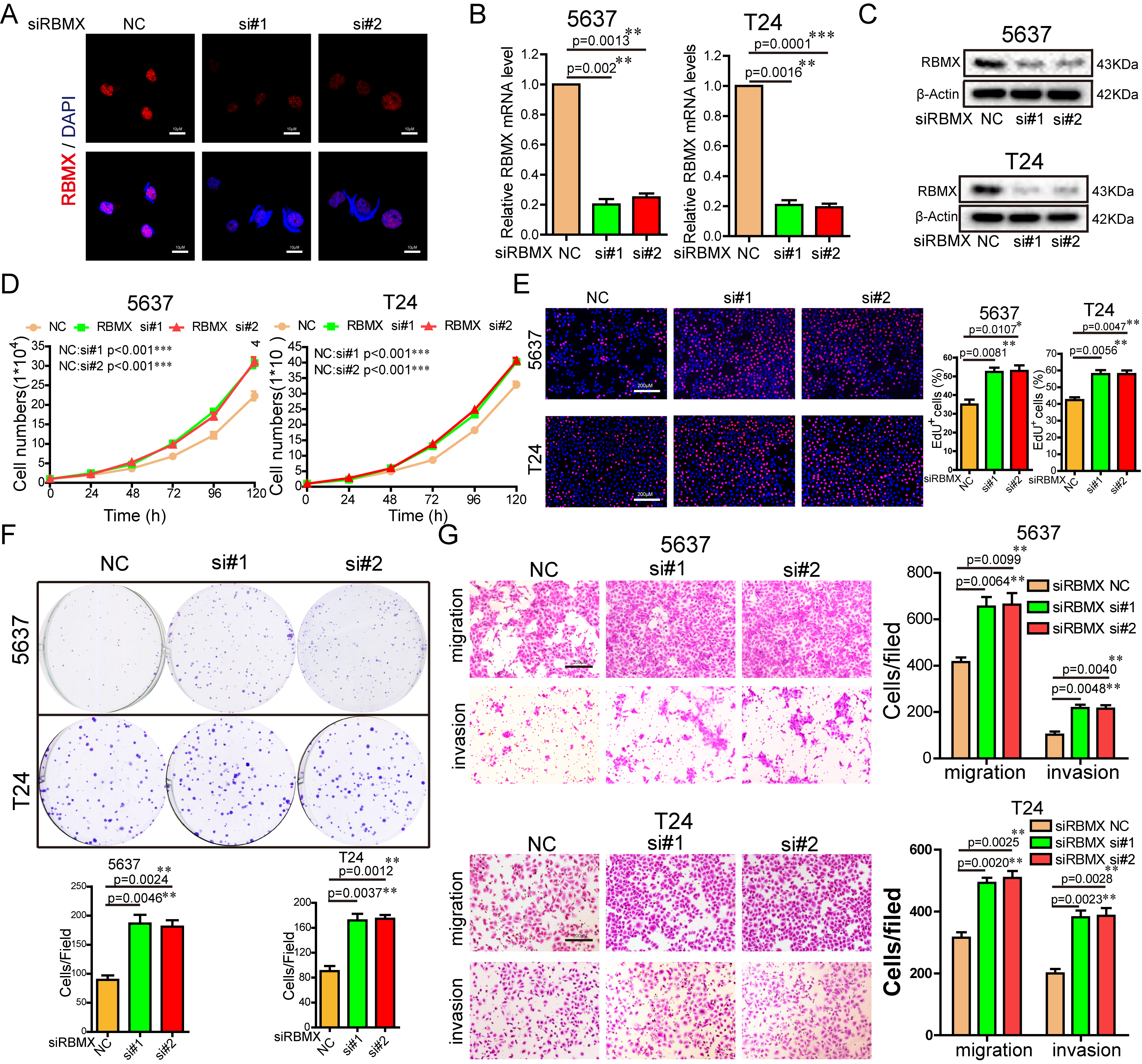

Supplement: Supplementary file 2 — Supplementary Figure 2 [file 41388_2021_1666_MOESM2_ESM.tif]

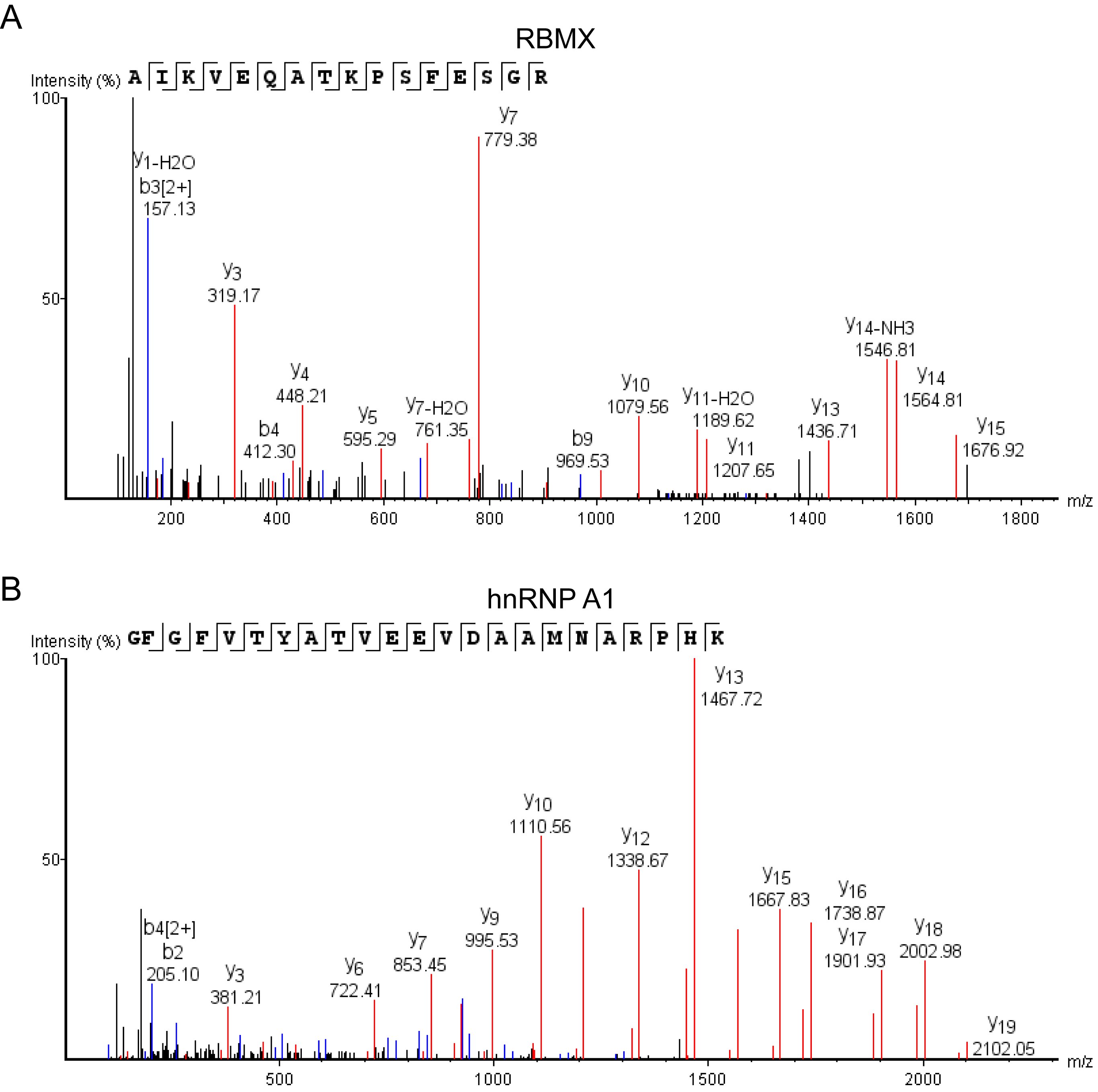

Supplement: Supplementary file 3 — Supplementary Figure 3 [file 41388_2021_1666_MOESM3_ESM.tif]

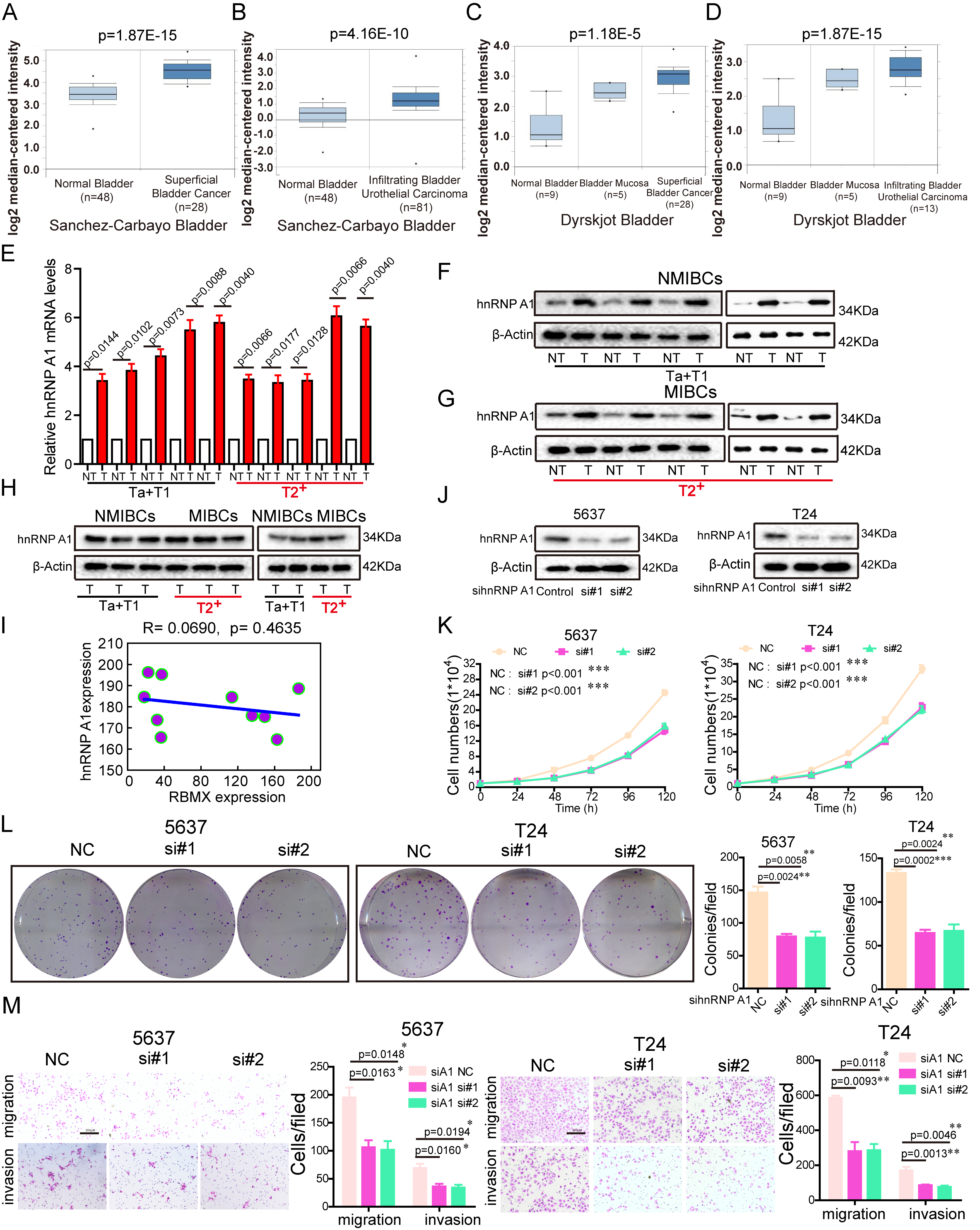

Supplement: Supplementary file 4 — Supplementary Figure 4 [file 41388_2021_1666_MOESM4_ESM.tif]

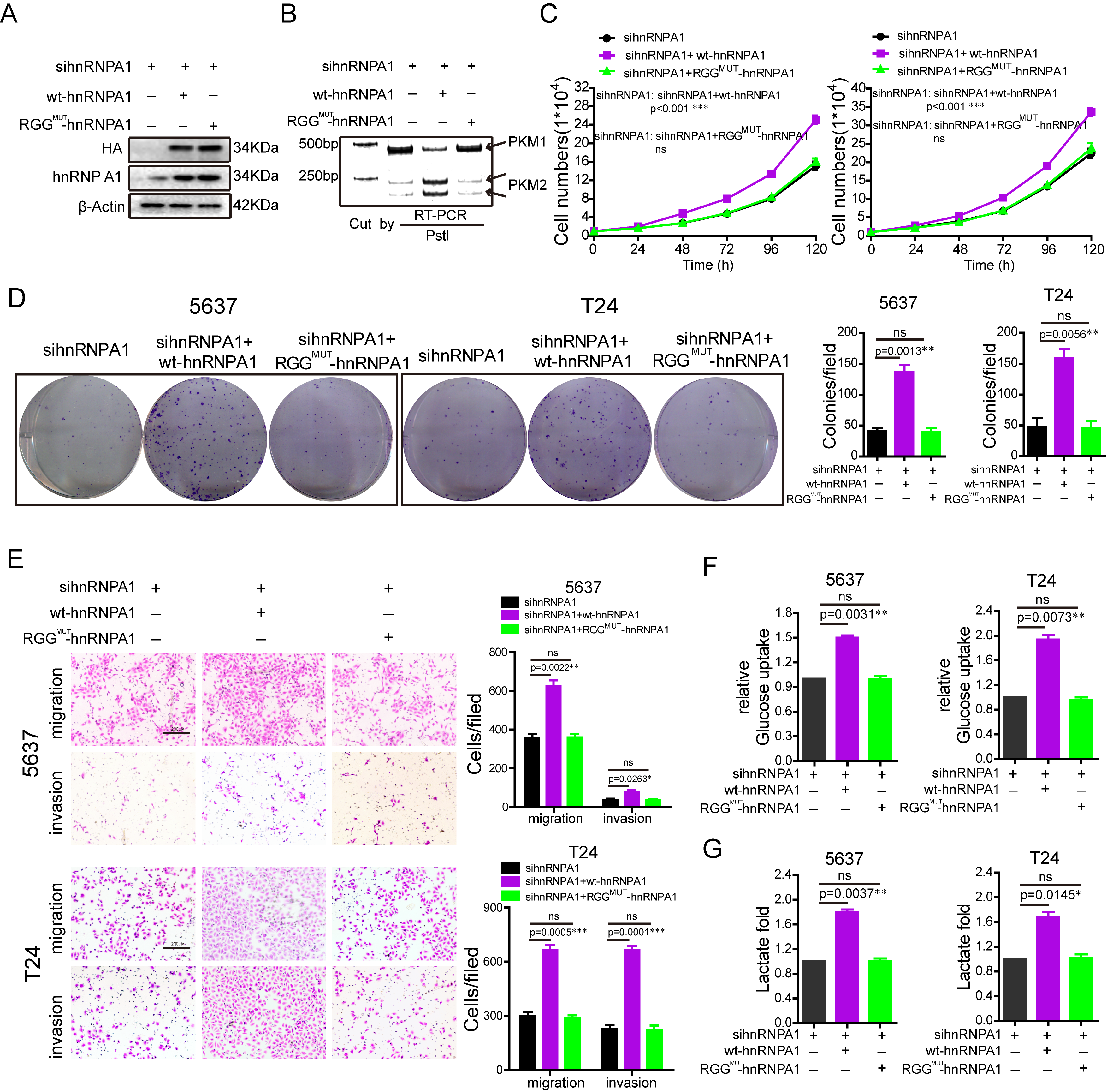

Supplement: Supplementary file 5 — Supplementary Figure 5 [file 41388_2021_1666_MOESM5_ESM.tif]

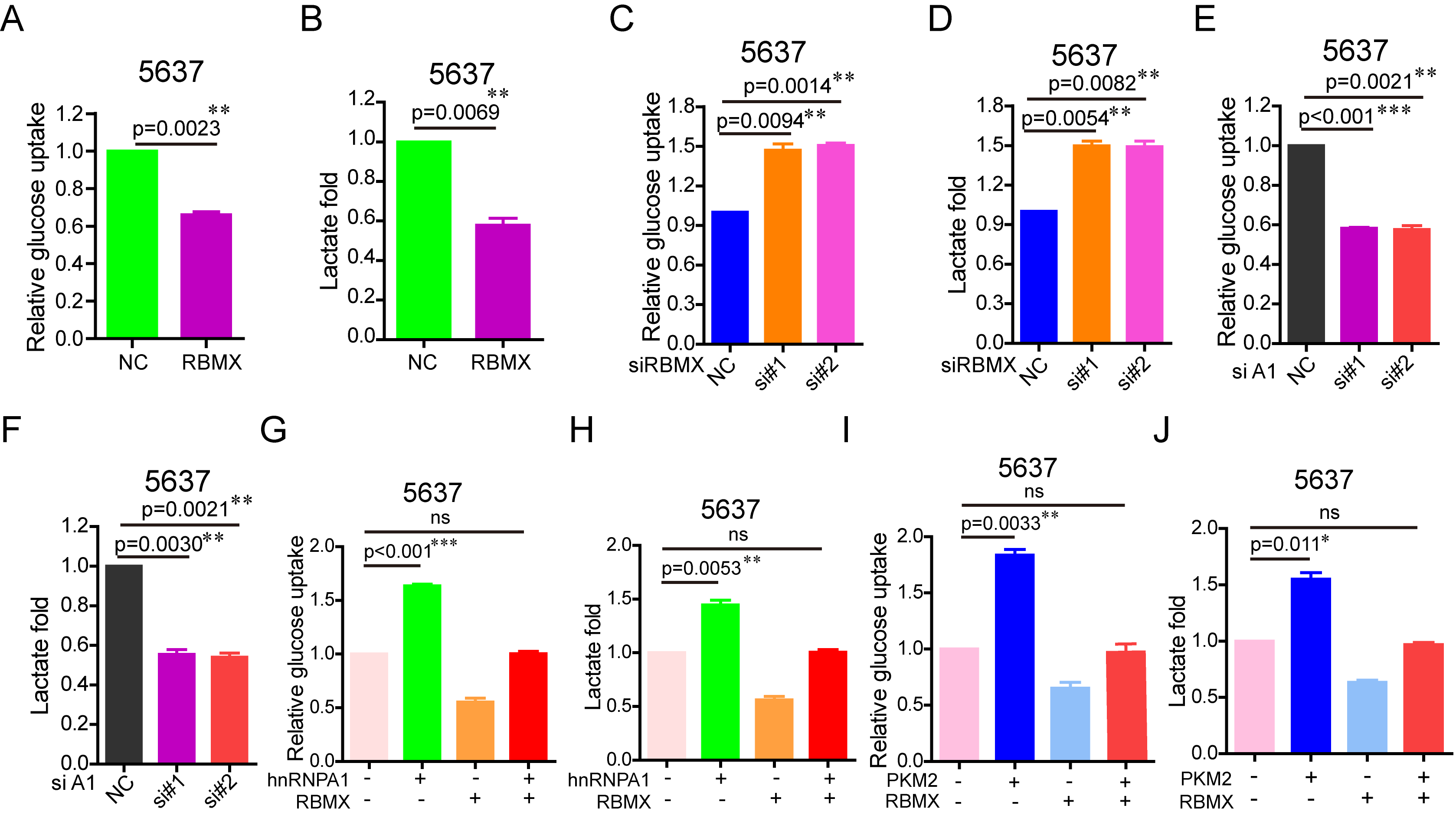

Supplement: Supplementary file 6 — Supplementary Figure 6 [file 41388_2021_1666_MOESM6_ESM.tif]
