# Supplementary material for: RBMX suppresses tumorigenicity and progression of bladder cancer by interacting with the hnRNP A1 protein to regulate PKM alternative splicing
Source: Oncogene. 2021 Feb 9;40(15):2635–50. doi: 10.1038/s41388-021-01666-z (PMC8049873; doi:10.1038/s41388-021-01666-z)
Supplement: Supplementary file 7 — Supplementary Figures Legends [file 41388_2021_1666_MOESM7_ESM.docx]

**Supplementary Figures Legends**

**Supplementary Fig. 1** Analysis of RBMX mRNA expression in the NMIBC tissues and MIBC tissues in the Blaveri, Sanchez-Carbayo and Stransky bladder data sets based on the Oncomine database.

**Supplementary Fig. 2 Silencing RBMX promoted the malignant phenotypes of the BCa cells.** **(A)** RBMX in the T24 cells was knocked down by siRNA. RBMX was immunostained using anti-RBMX antibodies. **(B-C)** RBMX silencing expression was determined in 5637 and T24 cells by qRT-PCR and western blot. **(D-G)** The effects of RBMX silencing on 5637 and T24 cell growth (D), proliferation (E), colony formation (F), migration and invasion (G) were examined. Data are presented as the means ±SD.

**Supplementary Fig. 3** The unique peptides of RBMX **(A)** and hnRNPA1 **(B)** were identified using mass spectrometry.

**Supplementary Fig. 4 Silencing hnRNP A1 suppressed the malignant phenotypes of the BCa cells. (A-D**) Analysis of hnRNP A1 mRNA expression in the NMIBC tissues, MIBC tissues, and normal bladder tissues based on the Oncomine database. **(E)** hnRNP A1 RNA levels in the NMIBC tissues (n=5), MIBC tissues (n=5), and matched adjacent NT tissues (NT) (n=10) were detected by qRT-PCR. **(F-H)** hnRNP A1 protein levels in the NMIBC tissues (n=5), MIBC tissues (n=5), and matched adjacent NT tissues (n=10) were detected by western blot. Data are presented as the means ± SD. **(I)** The protein level of hnRNP A1 negatively correlates with the level of RBMX by protein gray analysis. Pearson product moment correlation test, R=0.0690, P=0.4635. **(J)** After hnRNP A1 siRNAs were transfected into 5637 and T24 cells, the hnRNP A1 protein was detected by western blotting. **(K-M)** The effects of hnRNP A1 silencing on 5637 and T24 cell growth (K), colony formation (L), migration, and invasion (M) were determined. Data are presented as the means ±SD.

**Supplementary Fig. 5 RGG domain is the key domain of hnRNP A1 in cell function.** **(A)** After knocking down hnRNP A1, wt-hnRNP A1, RGGMUT-hnRNP A1 or wt-hnRNP A1 plasmid together with the RGGMUT-hnRNP A1 plasmid were transfected into T24 bladder cancer cells. **(B)** PKM splicing was performed using PstI. **(C-E)** After knocking down hnRNP A1, the hnRNP A1 wild type plasmid and RGG^MUT^-hnRNP A1 plasmid were transfected into 5637 and T24 cells. The cell growth (C), colony formation (D), migration, and invasion were determined (E). **(F-G)** The glucose uptake and lactate production were measured. Data are presented as the means ± SD.

**Supplementary Fig. 6** **RBMX inhibited aerobic glycolysis through hnRNP A1-dependent PKM splicing in the 5637 cells.** **(A-F)** Flag-RBMX plasmid (A, B), RBMX siRNAs (C, D) or hnRNP A1 siRNAs (E, F) were transfected into 5637 cells, and then, the glucose uptake and lactate production were measured. **(G-H)** Flag-RBMX, HA-hnRNP A1, or Flag-RBMX plasmid together with the HA-hnRNP A1 plasmid were transfected into 5637 cells, and then, the glucose uptake and lactate production were measured. **(I-J)** Flag-RBMX, HA-PKM2, or Flag-RBMX plasmid together with the HA-PKM2 plasmid were transfected into 5637 cells, and then, the glucose uptake and lactate production were measured. Data are presented as the means ±SD.
