# Supplementary material for: RBMX suppresses tumorigenicity and progression of bladder cancer by interacting with the hnRNP A1 protein to regulate PKM alternative splicing
Source: Oncogene. 2021 Feb 9;40(15):2635–50. doi: 10.1038/s41388-021-01666-z (PMC8049873; doi:10.1038/s41388-021-01666-z)
Supplement: Supplementary file 10 — Supplementary Table 3 [file 41388_2021_1666_MOESM10_ESM.docx]

**Supplementary Table 3. The primers used in this study**

| Primers name |  | Sequence (5’-3’) |
| --- | --- | --- |
| RBMX-Flag | Forward | CCCAAGCTTCAGGGCAGCGTCAAGAGTC(HindIII) |
|  | Reverse | CGCGGATCCTTACTTGTCATCGTCGTCCTTGTAGTCACAAGGTGATAGTTCTTCATTGAATCC(BamHI) |
| hnRNPA1-HA | Forward | CGGGGTACCACGTTCGTCAGCTTGCTCCTT |
|  | Reverse | CGGAATTCTTAAGCGTAATCTGGAACATCGTATGG GTAAAATCTTCTGCCACTGCCATAGC |
| hnRNP A1-HA MUT1 | Forward | CGGGGTACCACGTTCGTCAGCTTGCTCCTT |
|  | Reverse | CGGAATTCTTAAGCGTAATCTGGAACATCGTATGG GTAAGTTAAGTGGGCACCTGGTC |
| hnRNP A1-HA MUT2 | Forward | CGGGGTACCACGTTCGTCAGCTTGCTCCTT |
|  | Reverse | CGGAATTCTTAAGCGTAATCTGGAACATCGTATGG GTATGAAGCACTAGCCATCTCTTGCTT |
| hnRNP A1-HA MUT3 | Forward | CGGGGTACCACGTTCGTCAGCTTGCTCCTT |
|  | Reverse | CGGAATTCTTAAGCGTAATCTGGAACATCGTATGG GTATCCATTATAGCCATCCCCACTGC |
| hnRNP A1-HA MUT4 | Forward | CGGGGTACCATGCCAGGTGCCCACTTAACTGT |
|  | Reverse | CGGAATTCTTAAGCGTAATCTGGAACATCGTATGG GTAAAATCTTCTGCCACTGCCATAGC |
| hnRNP A1-HA RGG^mut^ | Forward | GTGGTGGTCGTGGAGGTGGTGCCGGTGGGAATGACAACTTCG |
|  | Reverse | CACCTCCACGACCACCACCAGCGTTTCCAGAACCACTTCGACCTC |
| PKM2-Flag | Forward | GAGGAAACTTCAGTGGTCGTGGTGGCGCTGGTGGCAGCCGTGGTGG |
|  | Reverse | GACCACTGAAGTTTCCTCCACGACCGGCGTTGTCATTCCCACCGGCA |
| GAPDH (RT-PCR) | Forward | GCAAATTCCATGGCACCGTCA |
|  | Reverse | TGGTCATGAGTCCTTCCACGAT |
| GAPDH (qRT-PCR) | Forward | CCTCTGACTTCAACAGCGACACC |
|  | Reverse | ACCACCCTGTTGCTGTAGCCAA |
| RBMX  (RT-PCR) | Forward | ATTTGACTTTCGCATTGCTT |
|  | Reverse | TGAGTAGCATACCCCAACCAC |
| RBMX  (qRT-PCR) | Forward | ATTTGACTTTCGCATTGCTT |
|  | Reverse | TGAGTAGCATACCCCAACCAC |
| PKM  (RT-PCR) | Forward | CTGAAGGCAGTGATGTGGCC |
|  | Reverse | ACCCGGAGGTCCACGTCCTC |
