# Supplementary material for: RBMX suppresses tumorigenicity and progression of bladder cancer by interacting with the hnRNP A1 protein to regulate PKM alternative splicing
Source: Oncogene. 2021 Feb 9;40(15):2635–50. doi: 10.1038/s41388-021-01666-z (PMC8049873; doi:10.1038/s41388-021-01666-z)
Supplement: Supplementary file 11 — Supplementary Materials [file 41388_2021_1666_MOESM11_ESM.docx]

**Supplementary Materials**

**Materials and methods**

**Tissue samples, experimental animal models, and cell culture**

BALB/c nude mice (n=6) were purchased from Hunan SJA Laboratory Animal Co., Ltd. (Hunan, China) to establish the BCa xenograft model and NOD-SCID mice (n=5) were also purchased from Hunan SJA Laboratory Animal Co., Ltd. (Hunan, China) to construct the BCa pulmonary metastasis model. All animal experiments were performed with the approval of Qingyuan People's Hospital Animal Care and Use Committee. HEK293T cells and T24 and 5637 human BCa cell lines were purchased from American Type Culture Collection (ATCC; Manassas, VA, USA). The HEK293T cells were maintained in DMEM (Invitrogen) containing 10% FBS and 1% penicillin-streptomycin. The 5637 and T24 cells were grown in 1640 medium (Invitrogen) containing 10% FBS and 1% penicillin-streptomycin. All cell lines in this study were confirmed to be mycoplasma negative and authenticated by short tandem repeat (STR) profiling before the beginning of the study.

**Quantitative reverse-transcription PCR (qRT-PCR)**

The purity and concentration were detected using a NanoDrop 2000 spectrophotometer (Thermo Fisher Scientific, USA). Approximately 2 µg of total RNA was reverse-transcribed into first-strand cDNA using the PrimeScript^TM^ RT reagent kit consisting of gDNA eraser (TaKaRa, Japan). qRT-PCR was conducted using the SYBR^®^ Premix Ex Taq^TM^ Ⅱ (Tli RNaseH Plus) kit (TaKaRa, Japan) and a Bio-Rad CFX system. Fold changes were calculated using the ΔΔCt method and glyceraldehyde 3-phosphate dehydrogenase (GAPDH) mRNA was used as a reference.

**Western blot analysis**

Cells or tissue samples were suspended in 1× SDS buffer and then sonicated. The supernatant of the lysate was collected by centrifugation and its concentration was determined by the BCA method. Equal amounts of lysate samples were separated using 10% SDS-PAGE, transferred to PVDF membranes, and then detected by an enhanced chemiluminescence (ECL) kit. The following primary antibodies were used: Flag (1:2000, 8146S, CST), RBMX (1:3000, EPR16038, Abcam), hnRNP A1 (1:2000, sc-32301, Santa Cruz), PKM1 (1:2000, 15821-1-AP, Proteintech), PKM2 (1:2000, 15822-1-AP, Proteintech), and β-actin (1:4000, sc-8432, Santa Cruz).

**Immunofluorescence**

Cells were fixed with 4% paraformaldehyde for 20 min, washed with cold TBS twice, and then permeabilized with 0.1% Triton X-100 for 10 min. After blocking with 3% BSA for 2 h, the cells were incubated overnight with the indicated primary antibodies (1:1000) at 4 °C. After washing with TBST, the cells were incubated with Alexa Fluor 488-labeled goat anti-mouse IgG (1:1000, A0428, Beyotime) or Cy3-labeled goat anti-rabbit IgG (1:1000, A0516, Beyotime) secondary antibody for 2 h at room temperature. Nuclei were stained with DAPI for 1 min. Finally, the cells were observed under a confocal microscope.

**Lentivirus production and the generation of stable cell lines**

Full-length RBMX was amplified using PCR and cloned into a pCDH plasmid (Invitrogen, USA) using TaKaRa Primer STAR Max DNA Polymerase. pCDH-RBMX, psPAX2, and pMD2.G plasmids were transfected together into HEK293T cells to produce lentiviruses. 5637 and T24 cells were transfected with an overexpression RBMX or empty lentivirus, and screened using 6 μg/mLpolybrene. For lung metastasis in vivo, the stable overexpression RBMX or empty lentivirus was transfected with a Luc-lentivirus (GenePharma, Suzhou, China) which expresses luciferase.

**Cell growth and proliferation assay**

5637 and T24 cells were transfected with the indicated plasmids or siRNAs for 48 h. For cell growth assay, each kind of cell was seeded in 24-well plates at a density of 1×10^4^ cells per well. The number of cells in each treatment group was counted after 24, 48, 72, 96, and 120 h. To examine the proliferation ability, each kind of cell was plated in 12-well plates at a density of 2×10^5^ cells per well. According to the manufacturer’s instructions, cell proliferation was assessed using an EdU kit (C10310-1, RiboBio, China). Finally, the EdU stained cells were visualized using a fluorescence microscope.

**Cell colony formation assay**

5637 and T24 cells were transfected with the indicated plasmids or siRNAs for 48 h. A total of 5637 cells were seeded onto 6-well plates and cultured at 37 ℃ for 10 days; T24 cells (5×10^2^) were plated in 6-well plates and incubated at 37 ℃ for 7 days. The colonies were fixed with 4% paraformaldehyde for 15 min and stained with 0.5% crystal violet solution for 20 min. Colony formation ability was quantified by counting the surviving colonies containing more than 50 cells.

**Cell migration and invasion assays**

Cell migration and invasion abilities were evaluated using the Transwell technique, which was performed in Transwell plates (8 μm pores; Corning, USA) with and without Matrigel Matrix (BD, USA), respectively. 5637 and T24 cells were transfected with the indicated plasmids or siRNAs for 48 h. For the migration assays, 1×10^5^ 5637 cells were plated and cultured for 36 h in Transwell plates. T24 cells (5×10^4^) were plated and cultured for 24 h in Transwell plates. For the invasion assays, Matrigel was added to the upper chamber and incubated at 37 ℃ for 1 h. Then, 2×10^5^ 5637 cells were plated and cultured for 72 h in Transwell plates and 1×10^5^ T24 cells were plated and cultured for 48 h in Transwell plates.

**In vivo xenograft tumor model**

For the subcutaneous tumor model, 1×10^6^ cells (T24/LV-RBMX or T24/LV-NC) in 100 μL of PBS were subcutaneously injected into the left and right flanks of 4- to 5-week-old female BALB/c nude mice. Four weeks later, these mice were euthanized, and the tumor weights and volumes were calculated. For the spontaneous lung metastasis model, mice were randomly assigned into two groups according to the random number table method. 2×10^6^ cells (T24/LV-RBMX-Luc or T24/LV-NC-Luc) in 100 μL of PBS were injected into the tail vein of 4- to 5-week-old female NOD-SCID mice. After 4 weeks, these mice were sacrificed and examined for tumor growth and metastasis in the lung using an in vivo imaging system (IVIS).

**Coimmunoprecipitation and mass spectrometry**

Briefly, two 10 cm dishes with HEK293T cells at 80% confluency were transfected with Flag tagged RBMX or a control plasmid for 48 h. The whole cell lysates were prepared using lysis buffer. Then, co-IP was performed using anti-Flag antibodies, and the immune complexes were purified on protein A/G agarose beads (Santa Cruz, USA). After five washes with PBS, the immune complexes were dissolved in 1×SDS buffer and separated in 10% SDS-PAGE gel. Then, the gels were subjected to a fast silver stain according to the kit manufacturer’s instructions (P0017S, Beyotime, China). The different gel bands and their corresponding negative gel bands were excised and digested using trypsin. The harvested peptides were detected using nano-LC-MS/MS (AB SCIEX TripleTOF 5600, USA).

**RNA affinity purification**

Briefly, 1 nmol of each biotin-labeled RNA was conjugated overnight to 100 μL of streptavidin-agarose beads (Sigma, USA) in 500 μL of binding buffer (10 mm tris-HCl, pH 7.5; 1 mm EDTA; and 2 M NaCl) at 4 °C. Next, the beads were washed three times with binding buffer and three times with buffer D [20 mM HEPES, pH 7.9; 20% glycerol; 100 mM KCl; 0.2 mM EDTA; 0.5 mM dithiothreitol (DTT)]. In addition, nuclear proteins in the T24 cells were extracted by a nuclear and cytoplasmic protein extraction kit (P0017S, Beyotime, China). Cellular nuclear proteins (500 μg) were incubated with RNA-streptavidin-agarose beads at 30 °C for 30 min. After protein and RNA binding, the beads were washed with buffer D three times and buffer D without glycerol three times and eluted using 1×SDS. The elution products were detected by western blot using anti-Flag, anti-HA, anti-RBMX or anti-hnRNP A1 antibodies.

**RT-PCR and PKM splicing assays**

Briefly, total RNA in the cell or tissue samples was extracted using TRIzol (Invitrogen, USA) reagent. After RNA reverse transcription, the PCR products of PKM were digested using PstI, and then the digested mixtures were separated by 8% non-denaturing PAGE.

**Measurement of glucose uptake and lactate production**

For the glucose uptake assay, 5637 and T24 cells were transfected with the indicated plasmids or siRNAs in 1640 medium. After 36 h of culture, the medium was replaced with 1640 medium without phenol red and with L-glucose for 8 h. The measurement of the glucose in different treatment groups was detected by a glucose colorimetric assay kit (K606-100, BioVision, USA) according to the manufacturer’s instructions. Lactate production was detected by a lactate colorimetric assay kit (K627-100, BioVision, USA). 5637 and T24 cells were transfected with the indicated plasmids or siRNAs in 1640 medium and incubated for 48 h, and the culture medium was collected at the indicated times. The culture medium was mixed with lactate assay buffer to a total volume of 50 μL/well in a 96-well plate. Then, 50 μL of reaction buffer was added to every well and incubated for 30 min at room temperature. Lactate production (OD_450nm_ values) was measured in a microplate reader.
